# Defects in the Outer Limiting Membrane Are Associated with Rosette Development in the *Nrl^−/−^* Retina

**DOI:** 10.1371/journal.pone.0032484

**Published:** 2012-03-12

**Authors:** Michael W. Stuck, Shannon M. Conley, Muna I. Naash

**Affiliations:** Department of Cell Biology, University of Oklahoma Health Sciences Center, Oklahoma City, Oklahoma, United States of America; University of Florida, United States of America

## Abstract

The neural retinal leucine zipper (*Nrl*) knockout mouse is a widely used model to study cone photoreceptor development, physiology, and molecular biology in the absence of rods. In the *Nrl^−/−^* retina, rods are converted into functional cone-like cells. The *Nrl^−/−^* retina is characterized by large undulations of the outer nuclear layer (ONL) commonly known as rosettes. Here we explore the mechanism of rosette development in the *Nrl^−/−^* retina. We report that rosettes first appear at postnatal day (P)8, and that the structure of nascent rosettes is morphologically distinct from what is seen in the adult retina. The lumen of these nascent rosettes contains a population of aberrant cells protruding into the subretinal space that induce infolding of the ONL. Morphologically adult rosettes do not contain any cell bodies and are first detected at P15. The cells found in nascent rosettes are photoreceptors in origin but lack inner and outer segments. We show that the adherens junctions between photoreceptors and Müller glia which comprise the retinal outer limiting membrane (OLM) are not uniformly formed in the *Nrl^−/−^* retina and thus allow protrusion of a population of developing photoreceptors into the subretinal space where their maturation becomes delayed. These data suggest that the rosettes of the *Nrl^−/−^* retina arise due to defects in the OLM and delayed maturation of a subset of photoreceptors, and that rods may play an important role in the proper formation of the OLM.

## Introduction

Development of the mammalian photoreceptor cell layer is characterized by the sequential restriction of competence of retinal progenitor cells to either rod or cone cells [1]. Cone precursor formation begins around embryonic day (E) 11 and continues to just after birth, while rod precursor formation begins around E12 and continues until ∼postnatal day (P) 8 [2]. Following terminal differentiation, all photoreceptor precursors express the transcription factors cone-rod homeobox (CRX) and orthodenticle homeobox 2 (OTX2) that are critical for establishing a photoreceptor cell fate [3], [4], [5], [6]. Photoreceptor precursors which express a threshold level of active neural retinal leucine zipper (*NRL*) adopt a rod cell fate, reinforced and maintained by the *NRL* target gene nuclear receptor subfamily 2, group E, member 3 (Nr2e3), while those below this threshold level adopt a cone cell fate [7], [8], [9].

The *Nrl* knockout mouse is a widely used model to study cone cell development and physiology in the absence of rods. In the wild-type (WT) adult mouse, rods outnumber cones by approximately 30∶1 [1]. Without NRL, rod cells all differentiate into functional cone-like cells which express cone-specific proteins [7]. Since *NRL* acts in post-mitotic cells, the total number of photoreceptors does not change in the *Nrl^−/−^* retina compared to the WT retina [1], [7]. Outer segments (OSs) in the *Nrl^−/−^* retina have flattened, stacked lamellae similar to WT cone OSs, but are shorter and have some abnormalities [7], [10]. Interestingly, the *Nrl^−/−^* retina exhibits undulations of the outer nuclear layer (ONL), commonly referred to as rosettes [7]. These rosettes form by an unknown mechanism and are prominent in the adult retina.

One of the known causes of rosette formation in other models is a defect in the retinal outer limiting membrane (OLM) [11]. The OLM is a series of adheren junctions between photoreceptors and neighboring Müller glia that serves, among other functions, to determine apical-basal polarity in photoreceptor cells [12]. Proper formation of the OLM is critical for photoreceptor maturation, function, and vision. Mutations of Crb1, an important component of the OLM, can cause severe blinding diseases in humans including Leber congenital amaurosis and retinitis pigmentosa [13], [14]. Importantly, rosette formation has been observed in instances of both genetic and chemical disruption of the OLM [15], [16]. Here we present results demonstrating that rosette formation in the *Nrl^−/−^* retina is associated with defects in the formation of the OLM and delayed maturation of some photoreceptors. Given that the primary defect in the *Nrl^−/−^ retina* is a lack of rods, these data suggest that rods are required for the proper formation of the OLM and proper organization of the ONL.

## Materials and Methods

### Animals

All experiments and animal maintenance followed protocols approved by the University of Oklahoma's Institutional Animal Care and Use Committee (IACUC), protocol number 10-090, and the guidelines set forth by the Association for Research in Vision and Ophthalmology (ARVO). The *Nrl^−/−^* mice were bred from founders generously provided by Dr. Anand Swaroop (National Eye Institute, Bethesda, MD). The *Rds^−/−^* mice were bred from founders generously provided by Dr. Neeraj Agarwal (University of North Texas Health Science Center, Fort Worth, TX). Animals of both genders were used and all animals were reared under cyclic lighting conditions (12 h L/D).

### Tissue Collection

After euthanasia, eyes were enucleated, the cornea was punctured, and eyes were placed in 4% paraformaldhyde in PBS for 2 hours. All eyes were collected between 11 am and 2 pm. After 2 hours, the cornea and lens of each eye was removed and the eye cups were returned to fixative for 2 additional hours. The eyes were cryoprotected in a sequential sucrose gradient and cryosectioned as described previously [17]. Briefly, eyes were embedded in frozen Shandon M-1 embedding matrix (Thermo Electron Corporation) and 10 µm or 25 µm sections were collected (Leica Cryostat CM3050-S).

### Immunohistochemistry

For immunohistochemistry (IHC), slides were treated with 100% methanol for 20 minutes at −20°C followed by a 2 minute immersion in a 1% solution of NaBH_4_ at room temperature. Slides were incubated in blocking solution for 2 hours (2% normal goat serum, 5% BSA, 1% fish gelatin, 0.5% triton X-100, in either HBSS or PBS). Primary antibodies were diluted in blocking solution and sections were incubated overnight at 4°C in a dark humid chamber. After washing, secondary antibodies were diluted in blocking solution and incubated with sections for one hour at room temperature. Slides were mounted using ProLong® Gold antifade reagent with DAPI (Invitrogen, Carlsbad, CA) prior to imaging. Sections were imaged using a Hamamatsu C-4742 camera through UPlanSApo objectives (Olympus, Tokyo, Japan) on an Olympus BX62 upright microscope equipped with a spinning disk confocal unit. Image analysis was performed using Slidebook v4 software (Olympus), and ImageJ [18].

### Rosette Quantification

Rosettes were quantified in 4–5 mice per genotype/age. Nascent and mature rosettes were counted across three full 10 µm thick central retinal sections (containing the optic nerve head) for each mouse and averaged to give a value for that individual animal. An individual rosette was scored as nascent if it contained a population of cells lacking subretinal IS/OS staining and mature if it did not contain any cells. Results were analyzed using two-way ANOVA for genotype and age.

### Antibodies

Primary antibodies were used as follows: rabbit anti-S-opsin (generated in-house and characterized previously [19]) diluted at 1∶1000; goat anti-S-opsin (H-17, Santa Cruz Biotechnology, Santa Cruz, CA) diluted at 1∶500; rabbit anti-recoverin (generously shared by Dr. James F. McGinnis, OUHSC [20]) diluted at 1∶5000; mouse monoclonal anti-BrdU (G3G4, the monoclonal antibody developed by Stephen J. Kaufman was obtained from the Developmental Studies Hybridoma Bank developed under the auspices of the NICHD and maintained by the University of Iowa, Department of Biology, Iowa City, IA) diluted at 1∶10; rabbit anti-B-catenin (C2206, Sigma-Aldrich, St. Louis, MO), diluted at 1∶1000; rabbit anti-ZO-1(Mid) (40–2200, Invitrogen, Carlsbad, CA) diluted at 1∶100; mouse monoclonal anti-ezrin (3C12, Abcam, Cambridge, MA) diluted at 1∶50; rabbit anti-mCAR-LUMIj (generously shared by Dr. Cheryl Craft, USC [19], [21], [22], [23]); rabbit anti-water channel aquaporin 4 (A5971, Sigma-Aldrich, St. Louis, MO) and rabbit anti-RDS-CT (generated in-house and characterized previously [24]) diluted at 1∶2000. Secondary antibodies were raised in donkey and were specific to goat, rabbit, or mouse and conjugated to either Alexa-488 or Alexa-555 (Invitrogen, Carlsbad, CA). Alexa-488-conjugated peanut agglutinin (Invitrogen, Carlsbad, CA) was applied at 1∶20 dilution during the secondary antibody incubation.

### TUNEL

TUNEL staining was accomplished using the *In Situ* Cell Death Detection Kit, TMR red (Roche, Indianapolis, IN) according to the manufacturer's specifications with the following modifications. Prior to using the kit protocol, the slides were incubated in 100% methanol for 20 minutes at −20°C. Following the completion of the TUNEL protocol, the slides were subjected to the IHC protocol described above for co-labeling experiments.

### BrdU

Mice underwent three intraperitoneal injections with a 10 µg/µl solution of BrdU (Sigma-Aldrich, St. Louis, MO) dissolved in PBS at a final dose of 50 µg/g body weight. Injections occurred at 4 pm, 12 am and at 8 am and tissue was collected 24 hours after the initial injection. Eyes were collected and processed as described above. Slides underwent IHC as described above but with the initial methanol incubation replaced by a 20 minute incubation in 10 mM citrate buffer (pH 6.0) at 95°C.

### Electron Microscopy

Transmission electron microscopy was performed as previously described [25], [26]. Briefly, eyecups were embedded in plastic and thin sections (600–800 Å) were collected, stained, and imaged using a JEOL 100CX electron microscope at an accelerating voltage of 60 kV.

## Results

### Nascent rosette morphology is distinct from adult rosette morphology

**Figure 1:**
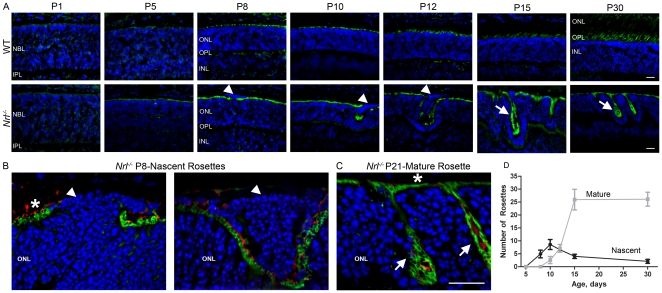
Development of rosettes. Retinal sections at the indicated ages were labeled with Alexa 488 conjugated peanut agglutin (PNA-green, **A–C**) and S-opsin (red, **B–C**). Small nascent rosettes (arrowheads) and mature rosettes (arrows) are observed. NBL: neuroblast layer, IPL: inner plexiform layer, ONL: outer nuclear layer, OPL; outer plexiform layer, INL: inner nuclear layer. Scale bar 20 µm **D**. Rosettes were counted in 3 sections/eye, 4–5 eyes per age/genotype. Shown are means ±SEM. Black line (squares)-nascent rosettes, Gray line (circles)-mature rosettes.

To begin our studies of the mechanism underlying rosette formation in the *Nrl^−/−^* retina, we established the time course of rosette development in this model. To visualize rosettes, we stained frozen sections of isolated eyes at the ages shown in (**Figure 1A**) using peanut agglutinin (PNA), a marker for the cone extracellular matrix, which decorates the area surrounding developing and mature cone ISs and OSs. At P1, PNA labeling is detected throughout the developing retina but quickly resolves into two discrete lines as the nuclear layers separate by P8 (**Figure 1A**). From P8 onwards, bright staining is detected in the developing IS/OS layer with fainter labeling detected around the developing cone terminals in the outer plexiform layer (OPL). At P8 in the WT retina, PNA labeling is detected in a straight line across the IS/OS layer, while in the *Nrl^−/−^* retina, breaks and infolding in the PNA labeled layer begin to be detected. These breaks highlight the initial formation of the rosettes (**Figure 1A** arrowheads). Initial rosette formation is observed at P8 in the *Nrl^−/−^* and these early (nascent) rosettes (arrowheads) have a different morphology than the mature rosettes seen in the adult retina (arrows).

To further study this morphological difference, we undertook higher magnification confocal imaging of retinal sections labeled with the cone IS/OS marker PNA (green) and the cone OS marker S-opsin (red). Mature rosettes appear as undulations of the ONL filled with the OSs of the surrounding photoreceptors (**Figure 1C** arrows). In contrast, the nascent rosettes in the developing retina contain not only early IS/OSs, but also a population of aberrant cells (**Figure 1B** arrowheads). In contrast to the photoreceptor nuclei outside rosettes which direct IS/OS into the subretinal space (**Figure 1B, C** asterisks), the aberrant cells inside the nascent rosettes lack IS/OS markers in the subretinal space (**Figure 1B**, arrowheads) suggesting that they either do not have OSs or that their OSs are misoriented. Some aberrant cells in nascent rosettes appear to be completely isolated from the surrounding ONL (**Figure 1B**, right) while in others, the aberrant cells appear to be contiguous with the ONL (**Figure 1B**, left). As the rosettes grow in size from P8 to P12, they appear to contain more aberrant cells (**Figure 1A**). Beginning between P12 and P15 however, the aberrant cells begin to disappear and the nascent rosettes are converted into mature rosettes which do not exhibit cell bodies in their lumen (**Figure 1C**, arrows). We observe that rosettes mature in a central to peripheral gradient consistent with the previously reported pattern of retinal development [27].

To understand further the timecourse of the transition from a retina with nascent rosettes to a retina with mature rosettes, we counted the number of rosettes in the *Nrl^−/−^* retina exhibiting nascent morphology (defined as those rosettes which contain aberrant cells lacking subretinal IS/OS staining) and the number of rosettes exhibiting mature morphology (defined as those which do not contain aberrant cells) in 10 µm thick central retinal cross sections containing the optic nerve. The number of nascent rosettes peaks at P10–12 after which it decreases significantly (**Figure 1D**, black lines). The decrease in nascent rosettes is accompanied by a significant, dramatic increase in the number of mature rosettes observed between P12 and P15 (**Figure 1D**, gray lines) consistent with maturation of rosettes and conversion from the nascent to the adult morphology during this time. Rosette formation is almost finished by P15; the number of mature rosettes observed in P15 retinas is not significantly different from the number observed in P30 retinas. The conversion of nascent nuclei-containing rosettes to mature “empty” rosettes occurs over time; at P15 many rosettes exhibit mature morphology while others still contain some aberrant cells, but by P30, no rosettes with nascent morphology are detected. Interestingly, the maximum number of mature rosettes (observed at P30) is approximately 3 fold higher than the maximum number of nascent rosettes (observed at P10), suggesting that each nascent rosette matures into multiple adult rosettes.

In the WT mouse, the majority of the subretinal space is filled with rod OSs with only a relatively small number of cone OSs. Since cone OSs are shorter and differently shaped than rods OSs [28], and in the *Nrl^−/−^* retina, all photoreceptors which would usually have a rod OS structure instead adopt a cone like structure, we wanted to rule out the possibility that the altered shape of the OSs (i.e. all cones) contributed to rosette development. To address this, we analyzed the timecourse of rosette development in an *Nrl^−/−^* retina which exhibits abnormal cone OS morphology. We chose the previously characterized *Rds^−/−^Nrl^−/−^* mouse model since it exhibits OSs that are functional but structurally deficient. The OSs of the *Rds^−/−^Nrl^−/−^* retina are simply open membranous sacs; they have no lamellae and no defined shape [29]. We did not detect any statistically significant differences in the number of rosettes or the timing of development between the *Nrl^−/−^ retina* and the *Rds^−/−^Nrl^−/−^* retina (**Figure S1**), suggesting that rosette morphology and development in the *Nrl^−/−^* retina is independent of OS structure.

**Figure 2:**
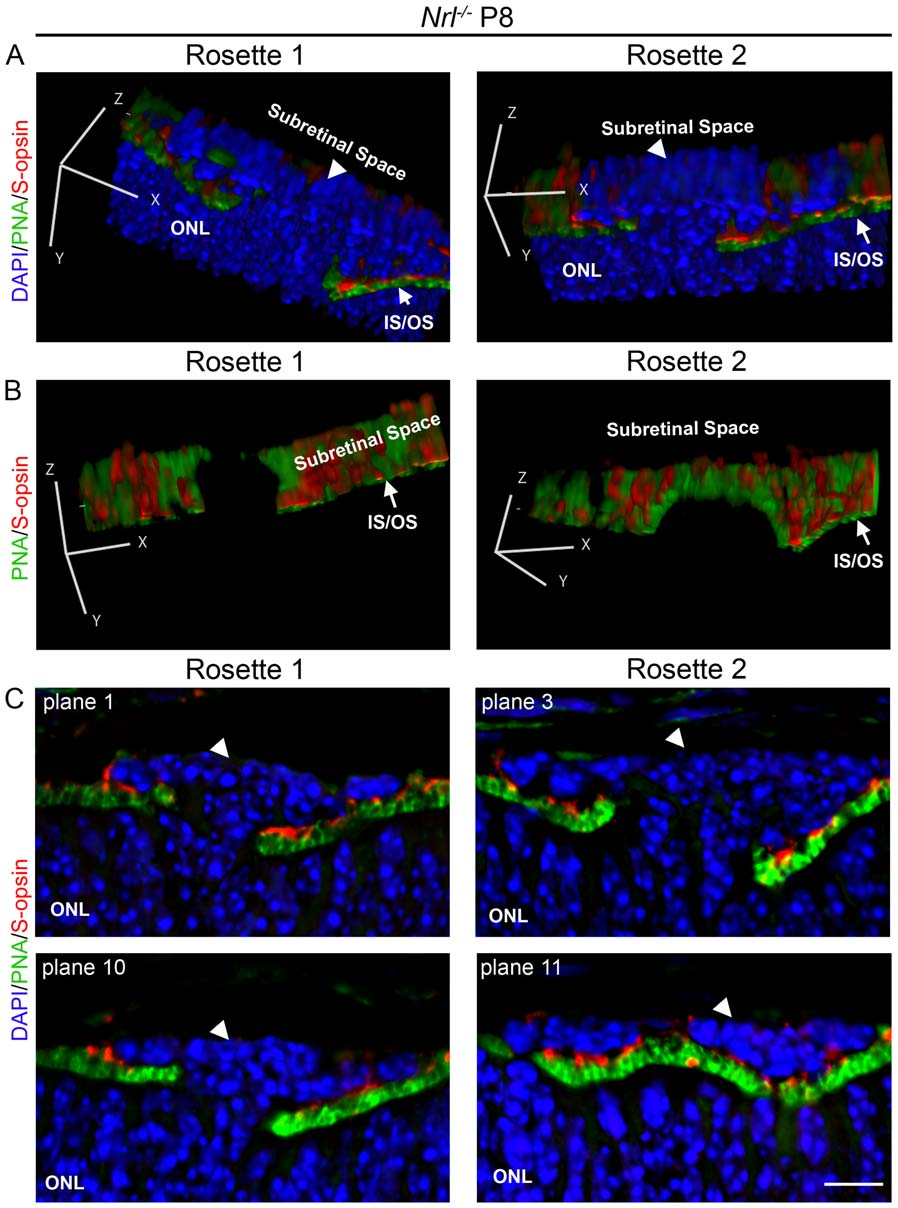
Three dimensional reconstruction of a P8 nascent rosette. Cone OSs were stained using S-opsin antibody (red) and cone extra cellular matrix was labeled with PNA (green) while nuclei were counterstained with DAPI (blue). Three dimensional reconstructions were created using ImageJ. **A**. Representative 3D reconstructions of two different nascent rosettes (arrowheads). **B**. The same two rosettes as in **A** are viewed with DAPI turned off and the viewing angle changed (refer to axes) to visualize the gaps through which the aberrant cells protrude. **C**. Selected single planes from the same two rosettes as **A–B**. ONL: outer nuclear layer, IS/OS: inner segments/outer segments Scale bar, 20 µm.

To gain a clearer understanding of the initial morphological characteristics of the nascent rosettes, we made three-dimensional reconstructions using a multi-plane confocal image taken at P8 and stained with PNA and antibodies against S-opsin. **Figure 2** shows two different representative rosettes in the left and right columns; panels **A** and **B** show two different angles of each rosette (refer to axes), one with DAPI and one without, while panel **C** shows two representative individual planes (out of ∼20) for each of the two rosettes. At P8, nascent rosettes are small enough that the structure can be reconstructed in a 25 µm image stack. In all cases we observed, the aberrant cells protrude out of the ONL, forming a mushroom-shaped clump of cells that is contiguous with the ONL (**Figure 2A**, arrowheads). The aberrant cells project through a gap in normal IS/OS staining, easily visualized when the DAPI channel is turned off (**Figure 2B**). The protruding cells spread out over the IS/OS labeling in the subretinal space. As a result of this spreading, in some planes, the aberrant cells appear to be fully enclosed by the IS/OSs lining the rosette (**Figure 2C**, plane 11-right and Figure 1B, right) while in other sections the DAPI-labeled nuclei of the aberrant cells are clearly contiguous with the DAPI-labeled nuclei of the ONL (**Figure 2C**, plane 3-right). Our observations suggest that all aberrant cells are contiguous with the ONL even in the larger nascent rosettes seen between P8 and P15, although this connection is not always observable in individual image planes.

### The aberrant cells are not due to abnormal late proliferation of photoreceptor cells

While previous work has shown that the adult *Nrl^−/−^* retina has a normal number of photoreceptor nuclei preceding subsequent degeneration [7], and cell birth in the retina is completed earlier than the onset of rosettes, the appearance of the aberrant cells at P8 suggested that there could be abnormal late cell proliferation occurring which is followed by a wave of cell death as the aberrant cells disappear and the rosettes mature. To determine whether the aberrant cells result from abnormal proliferation, *Nrl^−/−^* pups underwent a series of 3 intraperitoneal BrdU injections at 8 hour intervals beginning one day prior to the collection timepoint. Twenty-four hours after the initial injection, eyes were harvested co-labeled with S-opsin (to highlight rosettes-green) and anti-BrdU (red). Lack of BrdU labeling indicates that cells inside nascent rosettes are not actively dividing at either P8 or P10 (**Figure S2**, top). Similar results were obtained when the experiment was conducted at P12 and P15 (not shown). TUNEL assay was used to test whether the aberrant cells were being cleared through apoptosis as the rosettes matured (**Figure S3**). Apoptotic cells that are TUNEL positive (red) are observed within the INL of *Nrl^−/−^* and WT retinas until approximately P12 to P15 (**Figure S3**, arrows) consistent with normal retinal development, but no noticeable increase in apoptosis was observed inside the nascent rosettes (labeled with S-opsin-green) vs. outside at any time point (**Figure S3**, arrowheads).

### Rosette associated aberrant cells are underdeveloped photoreceptors

**Figure 3:**
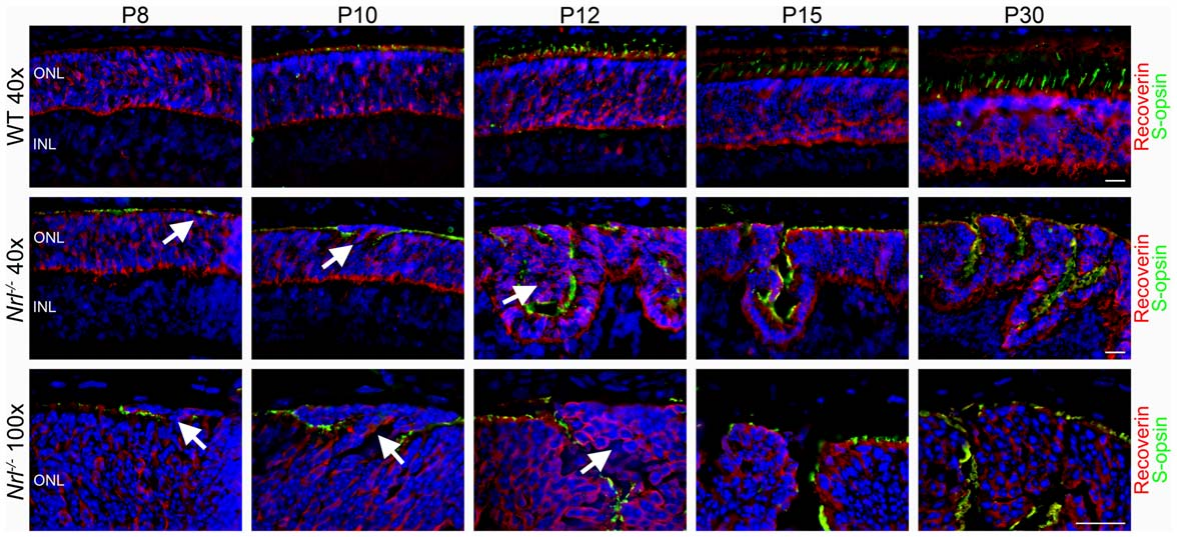
Rosette-associated aberrant cells are photoreceptors. Photoreceptor cell bodies were labeled using an antibody against recoverin (red) while OSs were stained using S-opsin (green) to aid in the identification of aberrant cells. All aberrant cells stain positive for recoverin (arrows) at all time points examined. ONL: outer nuclear layer, INL: inner nuclear layer. Scale bar, 20 µm.

Given that the population of cells found inside nascent rosettes is contiguous with the ONL and the appearance of their nuclei, we hypothesized that these cells were simply misplaced photoreceptor cells, despite their lack of IS/OS staining, rather than aberrantly migrating inner retinal cells or some other cell type. Therefore, sections were co-labeled with the photoreceptor cell body marker recoverin (red) and the cone OS marker S-opsin (green-**Figure 3**). The aberrant cells in the nascent rosettes stain positive for recoverin (**Figure 3**, arrows), regardless of age, confirming that they are committed photoreceptor cells.

**Figure 4:**
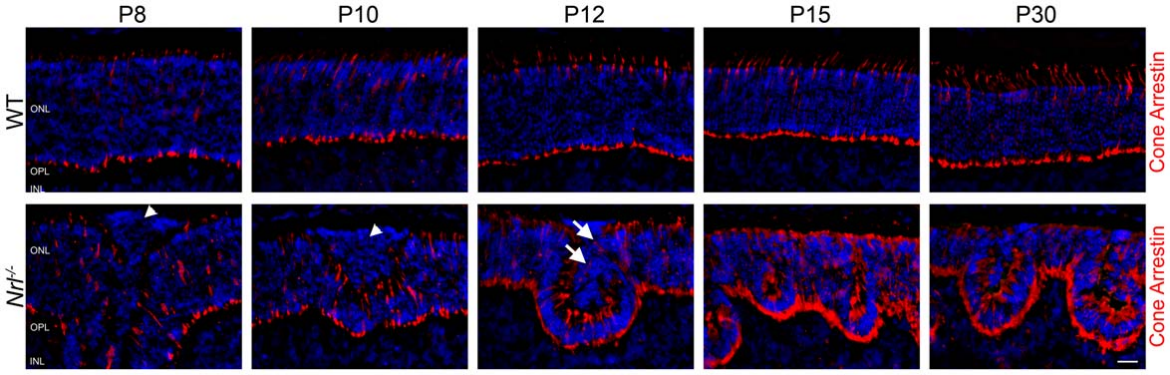
Early aberrant photoreceptors are not positive for cone arrestin. In order to further analyze the development of the cone photoreceptors in the *Nrl^−/−^* retina, cone photoreceptor cell bodies were stained using an antibody against cone arrestin (red). In the WT retina an even distribution of cone arrestin positive cells are observed throughout the retina and at all time points. Early aberrant photoreceptors at P8 and P10 lack significant staining for cone arrestin (arrowheads). At P12 cone arrestin staining is observed within the population of aberrant photoreceptors (Arrows). In the *Nrl^−/−^* retina, all photoreceptors are positive for cone arrestin at P15 and in the adult Scale bar, 20 µm.

To further understand the characteristics of these cells, we labeled retinal sections using an antibody against cone arrestin as a marker for mature cone photoreceptor cells (**Figure 4A**). In the WT retina, cone-arrestin labeled cone photoreceptors are spread evenly throughout the tissue and the number of positive cells remains relatively unchanged over time. Surprisingly, cone arrestin staining within the ONL in the *Nrl^−/−^* retina appeared similar to WT at early (P8–10) time points, i.e. not all cones in the *Nrl^−/−^* express cone arrestin at these ages. These results indicate that even though all the cells of the *Nrl^−/−^* are developing into cone-like cells they are not doing it at the same timecourse as the native cones of either the WT or the *Nrl^−/−^*. Furthermore, at P8 and P10, the aberrant photoreceptors were not positive for cone arrestin (**Figure 4**, arrowheads), confirming they are developmentally delayed and suggesting that they are not authentic cones. At P12 in the *Nrl^−/−^* retina, the cone arrestin staining became significantly more widespread compared to WT, and the aberrant photoreceptors begin to be cone arrestin positive (**Figure 4**, arrows), suggesting that these photoreceptors do mature, just at a delayed rate. By P15 all photoreceptor cells in the *Nrl^−/−^* appear to stain positive for cone arrestin.

**Figure 5:**
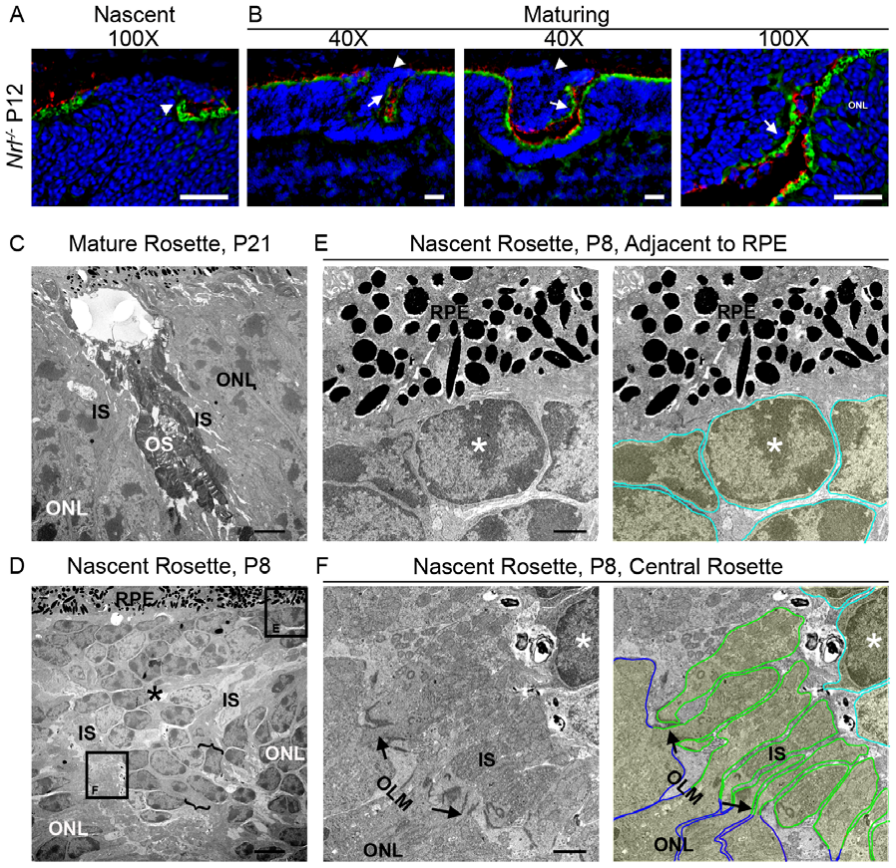
The aberrant photoreceptors in nascent rosettes do not exhibit IS/OSs. **A–B**. Transitional rosettes captured at P12 were labeled with PNA (green) and S-opsin (red). IS/OS labeling ends abruptly at the base of the aberrant photoreceptors in nascent rosettes (**A**-arrowhead), but can be seen along the edge of the populating of aberrant photoreceptors of maturing rosettes (**B**-arrow). Ultrastructural comparison of a mature (**C**) and nascent (**D**) rosette confirms the morphological characteristics observed using IHC including aberrant photoreceptors (**D–F**-asterisk). Black boxed regions in **D** are shown at higher magnification in **E–F**. **E**. Representative section of aberrant photoreceptors abutting the RPE. **F**. Representative section of aberrant photoreceptors inside the rosette. The ONL cells exhibit clear ISs protruding into the rosettes while the aberrant photoreceptors cells do not. Dark blue: ONL cell bodies; green: ONL IS; light aqua; aberrant photoreceptor cell bodies. Scale bar, A–B, 20 µm, C–D 4 µm E–F 1 µm.

Since our results suggest that aberrant cells are underdeveloped photoreceptors, we next asked whether they expressed IS/OS markers. We had previously observed that one of the distinguishing features of aberrant photoreceptors is a lack of ISs and OSs protruding into the subretinal space adjacent to the RPE (**Figure 1A–B**), but we could not rule out the possibility that the IS/OS of these cells were misoriented and pointed into the rosette. We therefore closely examined IS/OS labeling in nascent and maturing rosettes, which can both be seen in P12 sections. In the nascent rosettes (P8–10 and some at P12), aberrant photoreceptors are located in the subretinal space and IS/OS labeling ends abruptly at the edge of the population of aberrant photoreceptors (**Figure 5A**, arrowhead), and we observe no IS/OS protruding from the aberrant photoreceptors. However, at P12, as the rosettes begin to mature we observe some rosettes that exhibit a transitional morphology. The aberrant photoreceptors of these rosettes still lack ISs/OSs in the region adjacent to the RPE (**Figure 5B**, arrowheads) but start to exhibit PNA and S-opsin labeling protruding from the aberrant photoreceptors inside the rosette (**Figure 5B**, asterisks).

To further examine whether the aberrant photoreceptors exhibit ISs/OSs in nascent rosettes, we conducted ultrastructural analysis at P8 and P21. The ultrastructure of the mature rosettes is characterized by an orderly, layered morphology (**Figure 5C**). The outside of the rosette is defined by photoreceptor nuclei in the ONL. The next layer inwards comprises ISs, and the lumen of the rosette contains OSs that protrude from cells on all sides of the rosette (**Figure 5C**). In nascent rosettes (P8), the outside layer is still composed of the nuclei of the ONL, but instead of having additional concentric layers of ISs and OSs, the IS layer is interrupted by the aberrant photoreceptors which protrude into the lumen of the rosette (**Figure 5D**). Consistent with examination on the light level, the aberrant photoreceptors are contiguous with the ONL. The population of aberrant photoreceptors protrude through a relatively small gap in the IS layer (region between brackets in **Figure 5D**) and spread into the subretinal space toward the RPE (**Figure 5D**).

As we observed on the light level, ultrastructural examination shows that aberrant photoreceptors have no ISs or connecting cilia in the subretinal space and that the cell bodies of the aberrant photoreceptors come in direct contact with the apical surface of the RPE (**Figure 5E**, a magnified view of box E from **Figure 5D**). Similarly, **Figure 5F** (showing a magnified area of box F from **Figure 5D**) shows that in the nascent rosette ISs protrude from cells surrounding the rosette but not from aberrant photoreceptors. To help with visualization, the individual cells have been outlined and lightly highlighted in the right panels of **Figure 5E–F**. For each cell, the cell body is highlighted in blue (for ONL cells) or aqua (for aberrant photoreceptors) and the inner segments are outlined in green.

**Figure 6:**
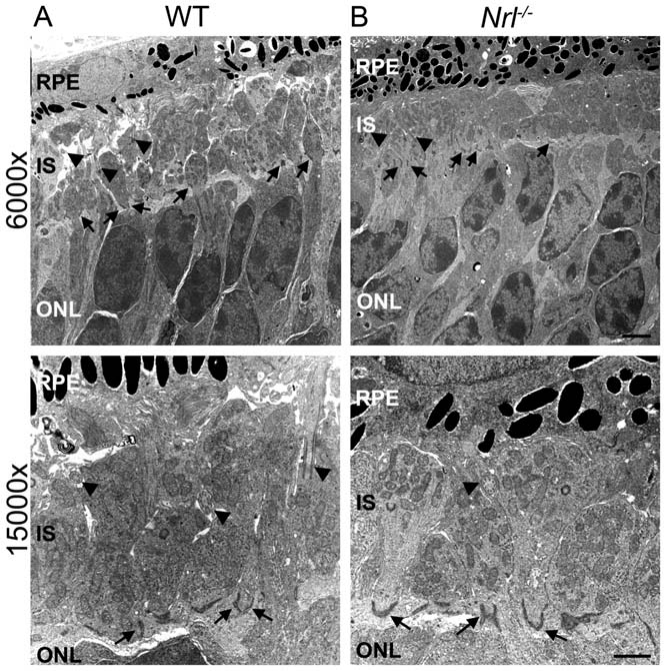
Ultrastructure of photoreceptor ISs of WT and *Nrl^−/−^* retinas at P8. The region between the RPE and the ONL at P8 in the WT (A) and *Nrl^−/−^* retina(B) is occupied by the well-formed ISs of photoreceptor cells and connecting cilia while OSs have not yet formed in the WT retina. Connecting cilia (arrowheads) can be observed in multiple orientations implying a lack of order in both WT and *Nrl^−/−^* retinas at P8 (A and B). Well-formed OLM junctions are also observed (arrows, A and B). Scale bar, 1 µm.

In contrast to the aberrant photoreceptors, *Nrl^−/−^* photoreceptors in an area outside the nascent rosettes appear similar to the WT. At P8, the region between the RPE and the ONL is filled predominantly with ISs since OSs are still undeveloped in both the WT and the *Nrl^−/−^* retinas. This region is not significantly different between the two genotypes (**Figure 6A–B**). Randomly oriented connecting cilia are observed in both the WT and the *Nrl^−/−^* retinas (**Figure 6A–B**, arrowheads) as are nicely formed ISs showing that in areas that lack rosettes, the photoreceptors are formed properly and are maturing in a regular fashion.

These data further support the idea that the aberrantly localized cells are underdeveloped photoreceptors which do not label with typical cone markers (arrestin, PNA, S-opsin) on the same timecourse as cells outside the rosettes, but as the retina ages, the cells appear to develop leading to the resolved mature rosette phenotype.

### RPE-photoreceptor associations are maintained in the *Nrl^−/−^* eye

**Figure 7:**
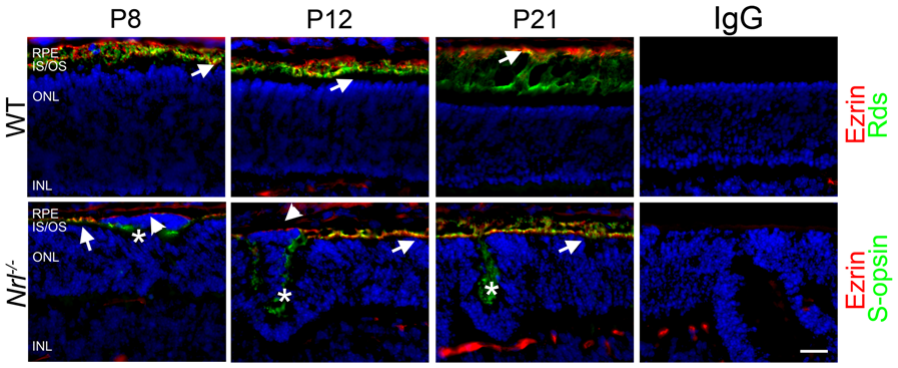
Associations between the RPE and photoreceptors are maintained in the *Nrl^−/−^* retina. RPE microvilli were labeled with ezrin antibody (red) and OSs were stained with Rds in WT or S-opsin antibody in *Nrl^−/−^* (green). Under normal conditions the RPE microvilli surround the apical portion of the photoreceptor OSs producing a yellow color (arrows). This association can be seen in both the WT and the *Nrl^−/−^* retinas. Ezrin staining does not extend into either the developing or the mature rosettes (asterisks). Directly between the aberrant photoreceptors and the RPE only ezrin labeling is observed (arrowheads). IS/OS: inner segments/outer segments, ONL: outer nuclear layer, INL: inner nuclear layer, RPE: retinal pigment epithelium. Scale bar, 20 µm.

Previous reports have demonstrated that retinal detachment and subsequent retinal folding/rosette formation can be associated with failure of the RPE microvilli to properly associate with the photoreceptor OSs [30]. To examine the possibility that photoreceptors in the *Nrl^−/−^* retina form abnormal associations with the RPE, we co-labeled retinal frozen sections from WT and *Nrl^−/−^* mice with antibodies against ezrin, a marker for the RPE microvilli (**Figure 7**, red), and Rds in WT or S-opsin in *Nrl* ^−/−^ as markers for OS (**Figure 7**, green). In the WT mouse, RPE microvilli surround the apical end of the OSs, visualized as yellow in **Figure 7** (arrows, top). Similarly, in the *Nrl^−/−^* retina, co-mingling of ezrin and S-opsin labeling in photoreceptors adjacent to the RPE indicates that the RPE microvilli can surround *Nrl^−/−^* cones (**Figure 7**, arrows, bottom). No RPE microvilli are detected inside rosettes at any age, note clear green staining only (S-opsin) inside rosettes (**Figure 7**, asterisks). In contrast, in the areas between the aberrant photoreceptors and the RPE, only ezrin staining is present (**Figure 7**, arrowheads) consistent with a lack of OSs on the aberrant cells. These results suggest that when cone OSs and RPE microvilli are present, associations are formed. When photoreceptors fail to properly orient their OSs towards the RPE no association is observed. These results imply that rosettes in the *Nrl^−/−^* are not formed as a result of any inherent inability of cone OSs to associate with the RPE. This point is further supported by the structural analysis and developmental timecourse of the *Rds^−/−^Nrl^−/−^* retina which shows no change in rosette structure or number in response to dramatic changes in OS structure (**Figure S1**).

### Defects in OLM formation are associated with rosette formation

**Figure 8:**
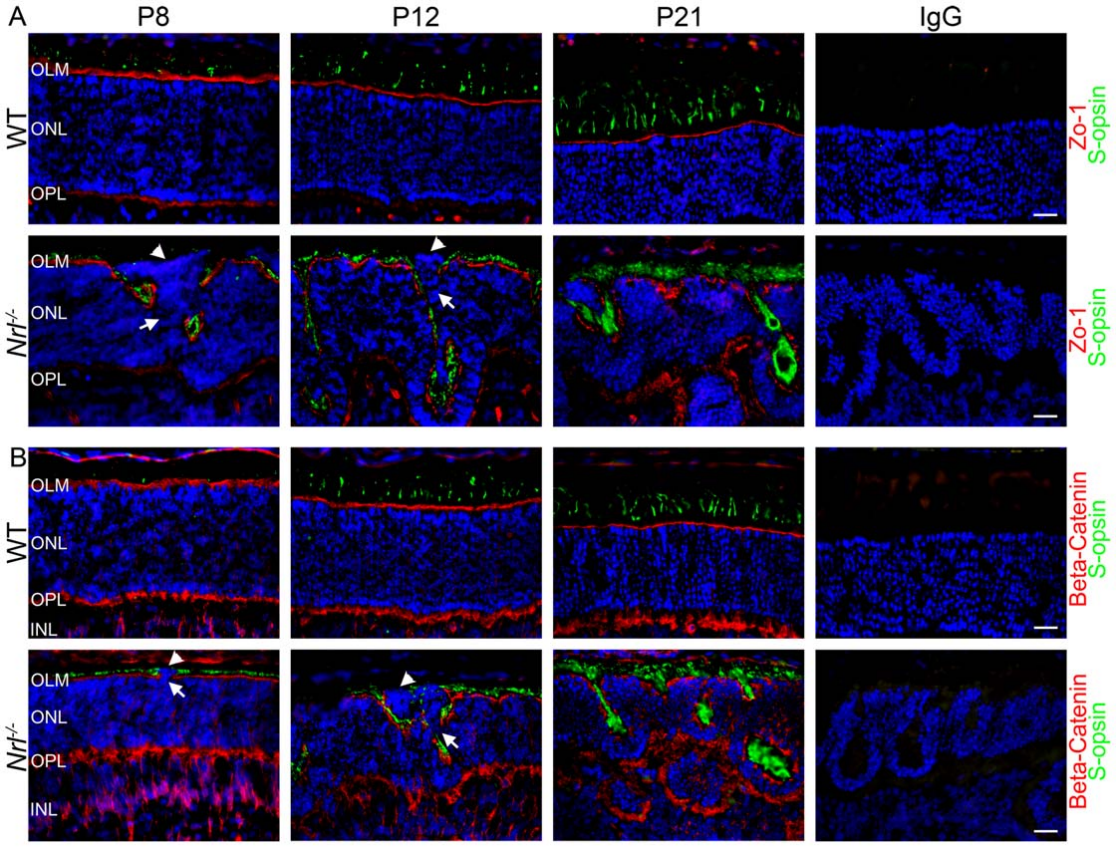
Defects in OLM formation are associated with rosette formation. To analyze the integrity of the OLM in the *Nrl^−/−^* retina, sections were stained with antibodies specific for Zo-1 and beta-catenin (red), components of the OLM junctions, and S-opsin (green) to label OSs. A. In the WT retina, the Zo-1 staining forms a straight continuous line. In the *Nrl^−/−^* retina, gaps in OLM staining (arrows) can be seen that correspond spatially and temporally with the appearance of nascent rosettes and aberrant photoreceptors (arrowheads). B. Beta-catenin staining can be seen at the OLM as well as in the OPL and INL. In the *Nrl^−/−^* retina, gaps in OLM staining (arrows) can be seen that correspond with the appearance of nascent rosettes and aberrant photoreceptors (arrowheads). OLM: outer limiting membrane, ONL: outer nuclear layer, INL: inner nuclear layer. Scale bar, 20 µm.

Improper photoreceptor layer development can also be due to defects in the formation of OLM [11], [16]. The OLM is composed of adherens junctions between photoreceptors and neighboring Müller glia, and it is possible that in the absence of rods, these junctions do not form properly. To test this hypothesis we labeled retinal sections with antibodies against two components of OLM junctions, Zo-1 and beta-catenin [31] (**Figure 8**, red). In the WT retina, the OLM is visualized as a solid line distinctly separating the ONL from the inner segment (IS) layer (**Figure 8A–B**, top). In the *Nrl^−/−^* retina, the OLM forms and regions lacking rosettes appear normal. However, in areas exhibiting nascent rosettes, a gap in the OLM can be seen (**Figure 8A**, arrows). The aberrant photoreceptors protrude through the gap in the OLM (**Figure 8A**, bottom, arrowheads) into the IS/OS region causing displacement of the surrounding nuclei and the formation of rosettes. A similar labeling pattern is observed when beta-catenin is used to decorate the OLM (**Figure 8B**). Furthermore, no OLM staining is detected surrounding the aberrant photoreceptors. In the adult *Nrl^−/−^* retina, the OLM appears more normal with labeling that lines the rosettes, clearly dividing the entirety of the ONL from the IS layer.

**Figure 9:**
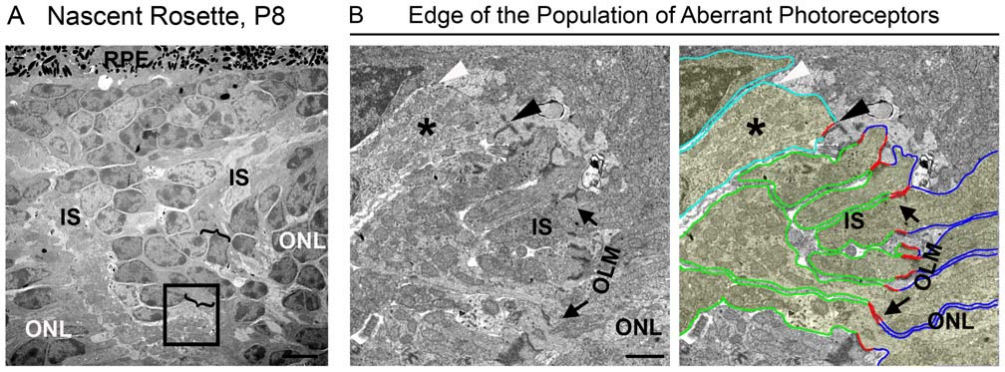
OLM Ultrastructure at the base of nascent rosettes. **A**. Ultrastructure of the nascent rosette from Figure 5D is presented here to orient the reader. **B**. Shown is magnified area from box. The first aberrant photoreceptor at the edge of the nascent rosette (asterisks) can be seen to form junctions with one neighboring cell (black arrowhead) yet fails to form junctions with other neighboring cells (white arrowhead). Dark blue: ONL cell bodies; red: OLM; green: ONL IS; light aqua; aberrant photoreceptors. Scale bar, A 4 µm B 1 µm.

Given the gaps in OLM that we see by immuno labeling with OLM markers, we wanted to examine the ultrastructure of the OLM where it terminates at the edge of the population of aberrant photoreceptors. This region was highlighted in brackets in **Figure 5D** and is shown again in **Figure 9A** for reference. A magnified view of this area is shown in **Figure 9B** (magnification of box from **Figure 9A**). For clarity, cells the right panel have been outlined; ONL cell bodies-blue, aberrant photorecptor cell bodies-aqua, ISs-green, OLM junctions-red. Each photoreceptor cell in the ONL that lines the rosette forms OLM junctions with its neighboring cells (**Figure 9**, red). However, this pattern of junctions and ISs is not preserved in the ring of cells surrounding the base of the population of aberrant cells. One such cell is labeled with an asterisk in **Figure 9**. The nucleus of this cell is found with the other aberrant photoreceptors, not in the ONL, and it is misoriented. The cell body protrudes from its nucleus towards the ONL and it forms an OLM junction with one ONL cell (black arrowhead, **Figure 9**), but on the other side forms no junctions (white arrowhead, **Figure 9**). Furthermore, although the cell body of this first aberrant photoreceptor protrudes past the nucleus, it does not exhibit an inner segment. This pattern of abnormal junctions and no ISs for the cells surrounding the edge of the population of aberrant photoreceptors is consistent among all observed rosettes at P8 and is also observed in nascent rosettes at P12. These observations imply that the aberrant photoreceptors retain some ability to form cell-cell adhesion complexes but nevertheless fail to form proper interactions with all neighboring cells. This likely leads to the abnormal protrusion of aberrant photoreceptors through the OLM and IS layer into the subretinal space.

**Figure 10:**
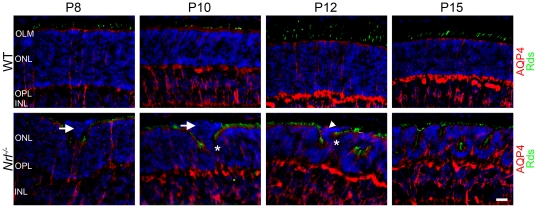
Early aberrant photoreceptor populations lack significant staining for Müller cell markers. In order to visualize the position of the Müller cells within the retina, sections were stained with antibodies specific for AQP4 (red) to label Müller cell bodies and S-opsin (green) to label photoreceptor OSs. Müller cell staining can be seen to end at a distinct line which corresponds to the OLM. Gaps in this line can be seen at the base of the aberrant photoreceptor populations (astrisks). Early aberrant photoreceptor populations at P8 and P10 lack prominent AQP4 staining, indicating a lack of Müller cell processes within these cell populations. AQP4 staining is observed within the aberrant photoreceptor populations at P12 (arrowhead). AQP4 staining pattern is indistinguishable from the adult at P15. ONL: outer nuclear layer, INL: inner nuclear layer, OPL: outer plexiform layer. Scale bar, 20 µm.

Since OLM defects are observed in the developing *Nrl^−/−^* retina it is important to establish the localization of the Müller glia cells that help form the OLM. In order to visualize the Müller cells we stained WT and *Nrl^−/−^* retinal sections with antibodies against the Müller cell marker aquaporin 4 (AQP4) [32], [33]. In the WT retina at all time points observed the AQP4 staining is visible from the inner limiting membrane to the OLM. At the OLM the AQP4 staining can be seen to end in a distinct line that corresponds to the position where the Müller glia cells take part in the formation of the OLM (**Figure 10**). In the *Nrl^−/−^* retina the Müller glia cells also terminate in a distinct line that corresponds to the location of the OLM (**Figure 10**). In the region where the population of aberrant photoreceptors connect to the ONL, there is no Müller cell labeling, consistent with the lack of OLM staining (**Figure 10**, asterisks). Furthermore, at P8 and P10 there is no Müller glia staining within the population of aberrant photorecptors (arrows) suggesting that the Müller cell processes have not successfully penetrated into this group of cells. At P12 however clear staining for Müller glia cell bodies is evident within the population of aberrant photoreceptors (**Figure 10**, arrowhead), further reinforcing the fact that the aberrant photoreceptors are in the process of finishing their maturation process at this time. By P15, Müller cell staining can be seen from the inner limiting membrane to the OLM.

## Discussion

**Figure 11:**
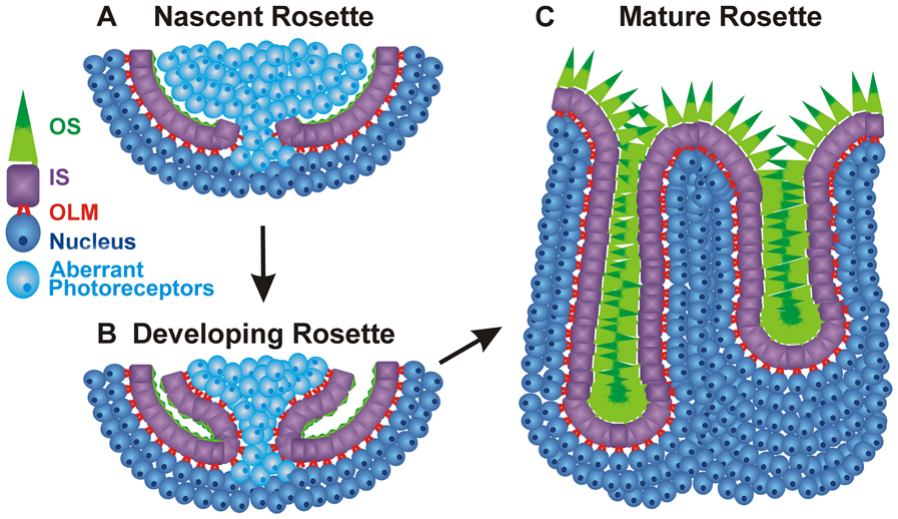
Model of rosette maturation. A. Nascent rosettes develop as abnormally migrating cell bodies (light blue) protrude through gaps in the OLM (red). These aberrant photoreceptors do not exhibit ISs or OLM junctions. B. As rosettes mature, the aberrant photoreceptors cells begin to develop ISs and OLM junctions. C. Mature rosette structure, in which the rosettes do not contain cell bodies.

Here we show that rosette formation starts at P8 in the *Nrl^−/−^* retina when photoreceptor nuclei begin to abnormally protrude into the subretinal space and induce ONL infolding (**Fig. 11A**). These aberrant photoreceptors are not actively dividing or undergoing apoptosis, rather they are underdeveloped and their abnormal localization is associated with gaps in the OLM. By P12–15 the aberrant photoreceptors are in the process of maturing, leading to the adult rosette phenotype lacking this population of underdeveloped cell bodies. This maturation is accompanied by the establishment of a largely continuous OLM, but overall ONL folding remains until retinal degeneration and thinning of the ONL begin later in the life of the animals [34].

While rosettes are seen in multiple genotypes and are a symptom of a variety of genetic retinal diseases, the root cause in each individual genotype is varied and it is important that each example of rosettes observed is examined independently [11], [30], [35]. Rosettes are reported in the naturally occurring Nr2e3 mutant mouse model (*rd7*) and were previously thought to be due to over-proliferation of S-cones [35]. Recent work however has demonstrated that the excess S-cones in the *rd7* mouse are due to a failure of a population of early born rod photoreceptor precursors to suppress cone specific genes [36]. While the reason for rosette formation was not directly studied in the *rd7*, both Nrl and Nr2e3 are within the same hierarchal developmental pathway, and our data here indicating that delayed maturation of a subpopulation of photoreceptors results in rosettes in the *Nrl^−/−^* suggests that a similar process could be at work in the *rd7* mouse model. Rosettes are also observed in the non-functional Crumbs 1 (CRB1) mutant mouse model (*rd8*) [11], [16]. Without CRB1, a component of adherens junctions, photoreceptors fail to properly form the OLM, a defect which leads to rosette formation [11], [16]. Finally, rosette formation has been shown to be associated with aberrant interaction between the RPE microvilli and photoreceptor OSs. For example, knockdown of the chloride intracellular channel 4 (CLIC4) has been shown to prevent proper association between OSs and the RPE microvilli, in turn causing localized retinal detachment and the formation of rosettes [30]. Taken together these results emphasize the need to approach the appearance of rosettes in each genotype and determine their individual root cause on a case by case basis.

The data presented here support the hypothesis that rosettes in the *Nrl^−/−^* mouse result from defects in the OLM and the presence of an underdeveloped population of photoreceptors. This observation is consistent with previous work by Dr. Janet Blanks' group showing that chemical disruption of Müller cells during retinal development in the WT mouse results in the same protrusion of photoreceptor cell nuclei into the subretinal space that we observe in nascent *Nrl^−/−^* rosettes [15]. Although chemical disruption of Müller cells results in gross alterations in Müller cell morphology, the resultant nuclei protrusion/rosette formation is consistent with the phenotype observed in cases of targeted OLM defects, e.g. in the case of CRB1 mutations [11], [16]. Here we show that Müller cell processes fail to extend into the population of aberrant photoreceptors found in nascent rosettes, consistent with the fact that Müller cells are required for the formation of the OLM.

These observations however, lead to the question, why does the cone-dominant retina exhibit defects in OLM formation? We have previously demonstrated that gross development and radial alignment of Müller cells in the *Nrl^−/−^* retina is normal [26], an expected outcome given that the *NRL* transcription factor is not involved in Müller cell differentiation. However, we here show that at early stages in development, Müller cells are not found in the regions where the underdeveloped photoreceptors are inducing rosette formation. While the exact cause of the OLM disruption in the *Nrl^−/−^* retina remains unclear, one possibility is that rods are required for proper OLM development. The adherens junctions which comprise the OLM consist of components expressed by both the photoreceptor cells and the Müller cells. Rods and cones may contribute different components, and in the absence of rod-mediated adherens junctions, the OLM does not form properly. We suggest that without rods, OLM formation is perturbed and some of the interactions which are required for proper photoreceptor positioning are lost. It is unclear why disruptions in the OLM resulting from a lack of rods would appear as localized disruptions, resulting in rosettes, instead of a global failure of OLM formation however, this localized rosette phenotype is consistent with observations from previous models [11], [15], [16]. For example, chemical disruption of the Müller cells and genetic disruption of the OLM (*rd8*) would both be expected to cause defects in the entire OLM, but actually result in a localized disruption in the OLM similar to what we observe here in the *Nrl^−/−^*retina [11], [15], [16].

In addition to being mislocalized, initially the aberrant photoreceptors are underdeveloped compared to other photoreceptors; they do not exhibit staining typical of ISs/Oss, no ISs/OSs are observed on the ultrastructural level and they lack important markers of development such as cone arrestin. This phenotype is likely tied to the observed OLM defect as OLM components and regulators such as CRB1 are critical for the development of photoreceptor cell polarity [12]. In addition, mutational studies have shown that Crumbs genes play a role in regulating the IS membrane size in photoreceptor cells and have been shown to impact cilia formation in renal epithelial cells [37]. The lack of proper cell-cell adhesions between the aberrant photoreceptors and their neighboring cells may perturb their ability to determine apico-basal polarity and likewise inhibit their ability to properly develop ISs and OSs.

Our results suggest that the aberrant photoreceptors found inside nascent rosettes do mature with time, i.e. they eventually form cell-cell junctions, IS, and OSs, and thus contribute to the growth of a mature rosette (**Figure 11**). Our data on the variety of rosette phenotypes seen at P12 support this idea. This “delayed-maturation” hypothesis would result in multiple mature rosettes for every nascent rosette (**Figure 11**), and is consistent with our data showing that the number of mature rosettes in the adult *Nrl^−/−^* is ∼3-fold higher than the peak number of nascent rosettes.

Critically, our findings are relevant to many retinal diseases. Defects in specific OLM components have been previously associated with retinal degenerations, but our data here, indicating that the absence of rods results in OLM abnormalities, may further our understanding of the mechanisms underlying rod-cone degenerations. Many diseases which affect rods, such as retinitis pigmentosa, subsequently lead to the loss of cone cells [38]. This trend is also observed in animal models; for example in VPP mice carrying rhodopsin mutations and exhibiting a retinitis pigmentosa phenotype, rapid loss of rod cells is followed by a slow degeneration of cone photoreceptor cells [39]. In these cases the loss of rod cells is often due to a mutation in a rod specific protein (such as rhodopsin) but the precise mechanism is poorly understood. Recent data suggests the loss of rods leads to a secondary loss of cones due to a combination of retinal detachment, oxidative stress and activation of microglia [34]. Our data show that rods are critical for proper OLM formation and maintenance which suggests that in addition to these factors another mechanism underlying secondary cone degeneration may be OLM defects.

These data may also be relevant for the development of effective cell-based therapies for retinal degenerations. It has been shown that when chemical disruption of the OLM is used in conjunction with cell therapy in WT mice, greater integration of the transplanted photoreceptor cells occurs [40]. Our data suggest that OLM defects may be a part of the normal pathology of a variety of retinal diseases in cases where rods are lost. This facet should be taken into account when considering the optimal experimental design and timing of delivery for cell therapy.

Here we present the first report demonstrating that the *Nrl^−/−^* retina exhibits defects in the OLM, and that this defect is associated protrusion of underdeveloped photoreceptors into the subretinal space leading to rosette formation. Even though the OLM defect is detected for only a short window during development of the mouse retina, it results in rosettes a persistent rosette phenotype. Importantly these data suggest that rod cells (which are absent in the *Nrl^−/−^* retina) are required for proper OLM formation.

## Supporting Information

Figure S1 **Rosette development is not different in the** ***Rds^−/−^Nrl^−/−^*** **compared to the** ***Nrl^−/−^*** **.** Left-Retinal sections at the indicated ages were labeled with Alexa 488 conjugated peanut agglutinin. Small nascent rosettes (arrowheads) and mature rosettes (arrows) are observed. NBL: neuroblast layer, IPL: inner plexiform layer, ONL: outer nuclear layer, OPL; outer plexiform layer, INL: inner nuclear layer. Scale bar 20 µm Right-Rosettes were counted in 3 sections/eye, 4–5 eyes per age/genotype. Shown are means ±SEM. Black lines-nascent rosettes, Gray lines-mature rosettes.
(TIF)

Figure S2 **Aberrant cells are not due to abnormal proliferation of photoreceptor cells.** Mice were treated with BrdU over a 24 hour period prior to tissue collection. Retinal sections were labeled using antibodies against BrdU (red) and cone S-opsin (green). A. None of the aberrant photoreceptors incorporate BrdU, indicating that these cells are not undergoing DNA replication and cell division. B. BrdU positive (dividing cells-arrows) are observed in the peripheral retina, and are shown as a positive control. IS/OS: inner segments/outer segments, ONL: outer nuclear layer, INL: inner nuclear layer. Scale bar, 20 µm.
(TIF)

Figure S3 **Aberrant photoreceptors are not cleared through apoptosis.** A. TUNEL staining (red) was performed and followed by staining of OSs using S-opsin antibody (green). TUNEL positive cells can be seen in the INL as part of normal retinal development (arrows). All aberrant photoreceptors are negative for TUNEL staining at all time points examined (arrowheads). As previously noted, at P15 both nascent rosettes (containing aberrant photoreceptors) and mature rosettes are observed. We hypothesized that aberrant photoreceptors at this timepoint would be the most likely to be undergoing apoptosis if cell death is the mechanism underlying transition from the nascent rosette phenotype to the mature rosette phenotype. However, we did not detect any apoptotic cells in the nascent rosettes remaining at P15 (asterisks). ONL: outer nuclear layer, INL: inner nuclear layer. Scale bar, 20 µm.
(TIF)
