# Supplementary figures and images for: Defects in the Outer Limiting Membrane Are Associated with Rosette Development in the Nrl−/− Retina
Source: PLoS One. 2012 Mar 12;7(3):e32484. doi: 10.1371/journal.pone.0032484 (PMC3299663; doi:10.1371/journal.pone.0032484)

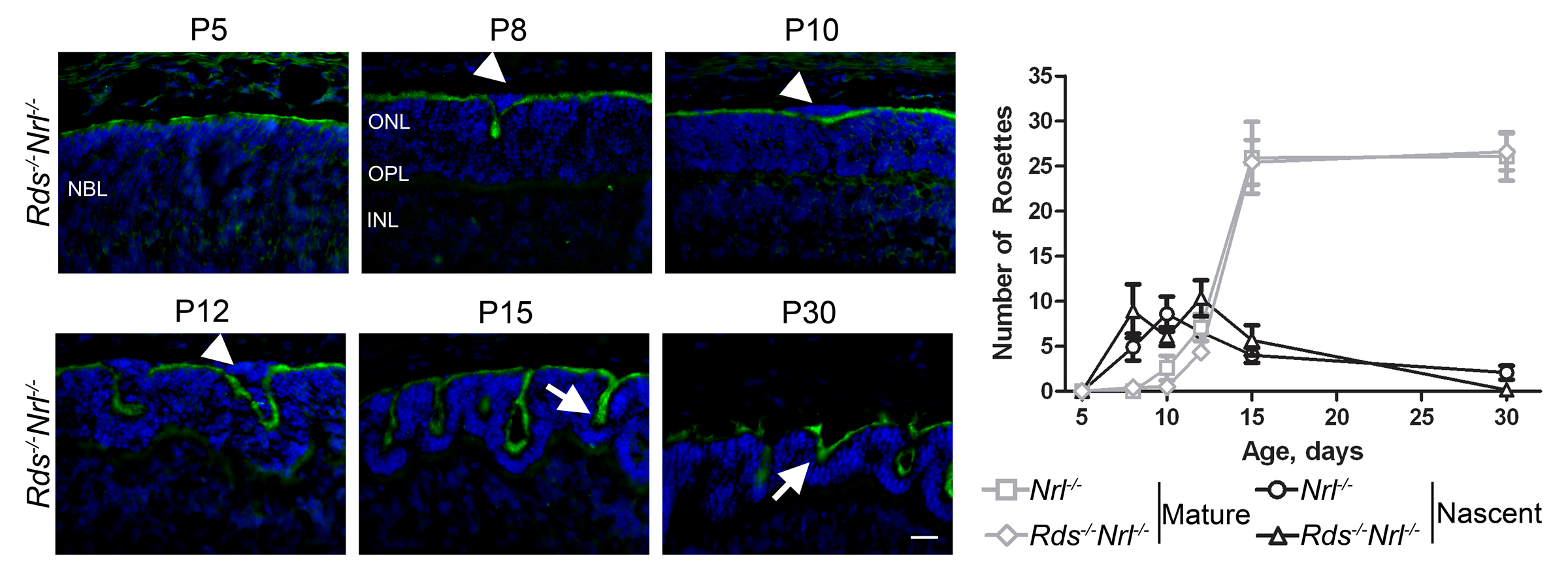

Supplement: Figure S1 — Rosette development is not different in the Rds−/−Nrl−/− compared to the Nrl−/− . Left-Retinal sections at the indicated ages were labeled with Alexa 488 conjugated peanut agglutinin. Small nascent rosettes (arrowheads) and mature rosettes (arrows) are observed. NBL: neuroblast layer, IPL: inner plexiform layer, ONL: outer nuclear layer, OPL; outer plexiform layer, INL: inner nuclear layer. Scale bar 20 µm Right-Rosettes were counted in 3 sections/eye, 4–5 eyes per age/genotype. Shown are means ±SEM. Black lines-nascent rosettes, Gray lines-mature rosettes. (TIF) [file pone.0032484.s001.tif]

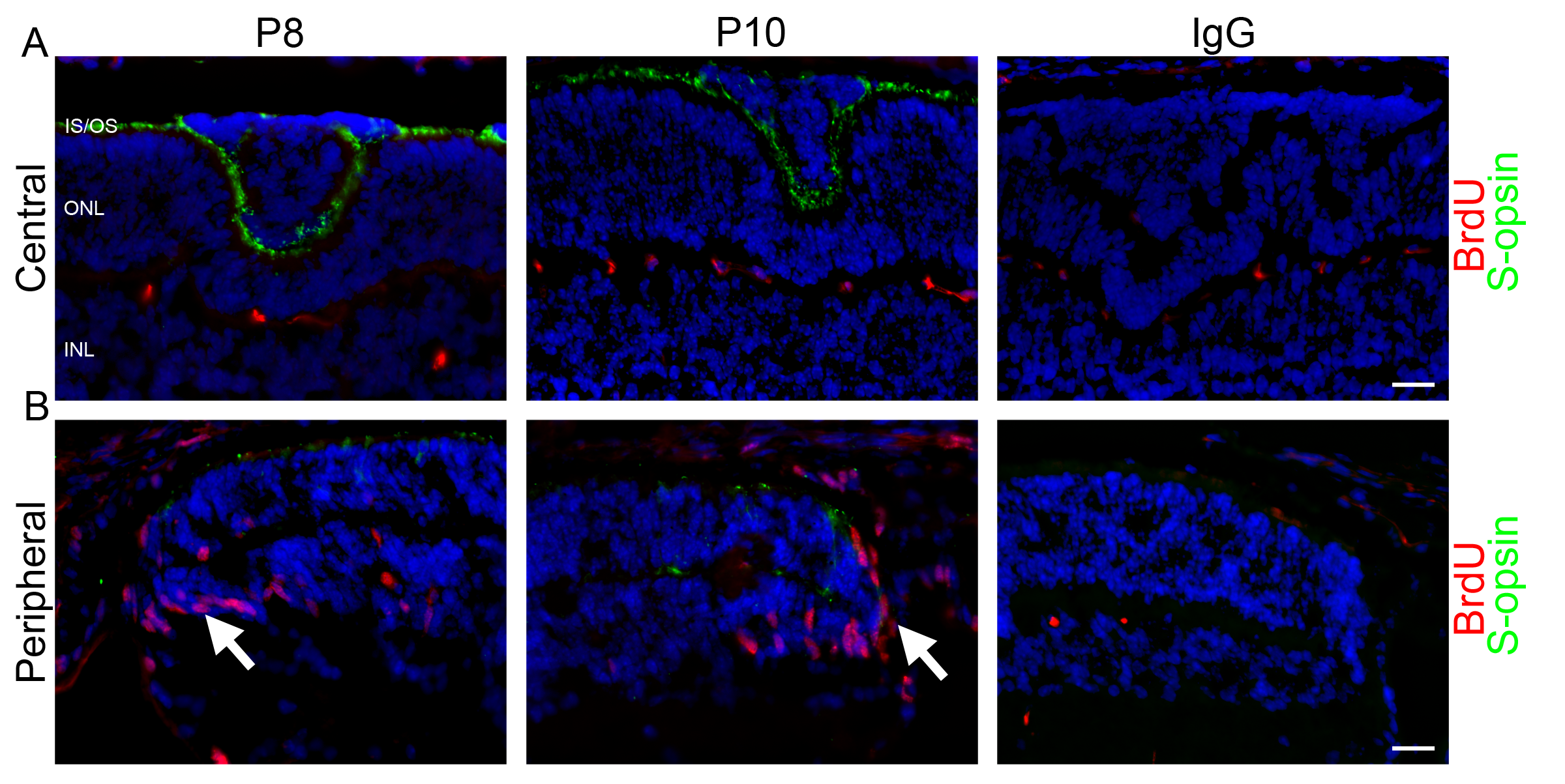

Supplement: Figure S2 — Aberrant cells are not due to abnormal proliferation of photoreceptor cells. Mice were treated with BrdU over a 24 hour period prior to tissue collection. Retinal sections were labeled using antibodies against BrdU (red) and cone S-opsin (green). A. None of the aberrant photoreceptors incorporate BrdU, indicating that these cells are not undergoing DNA replication and cell division. B. BrdU positive (dividing cells-arrows) are observed in the peripheral retina, and are shown as a positive control. IS/OS: inner segments/outer segments, ONL: outer nuclear layer, INL: inner nuclear layer. Scale bar, 20 µm. (TIF) [file pone.0032484.s002.tif]

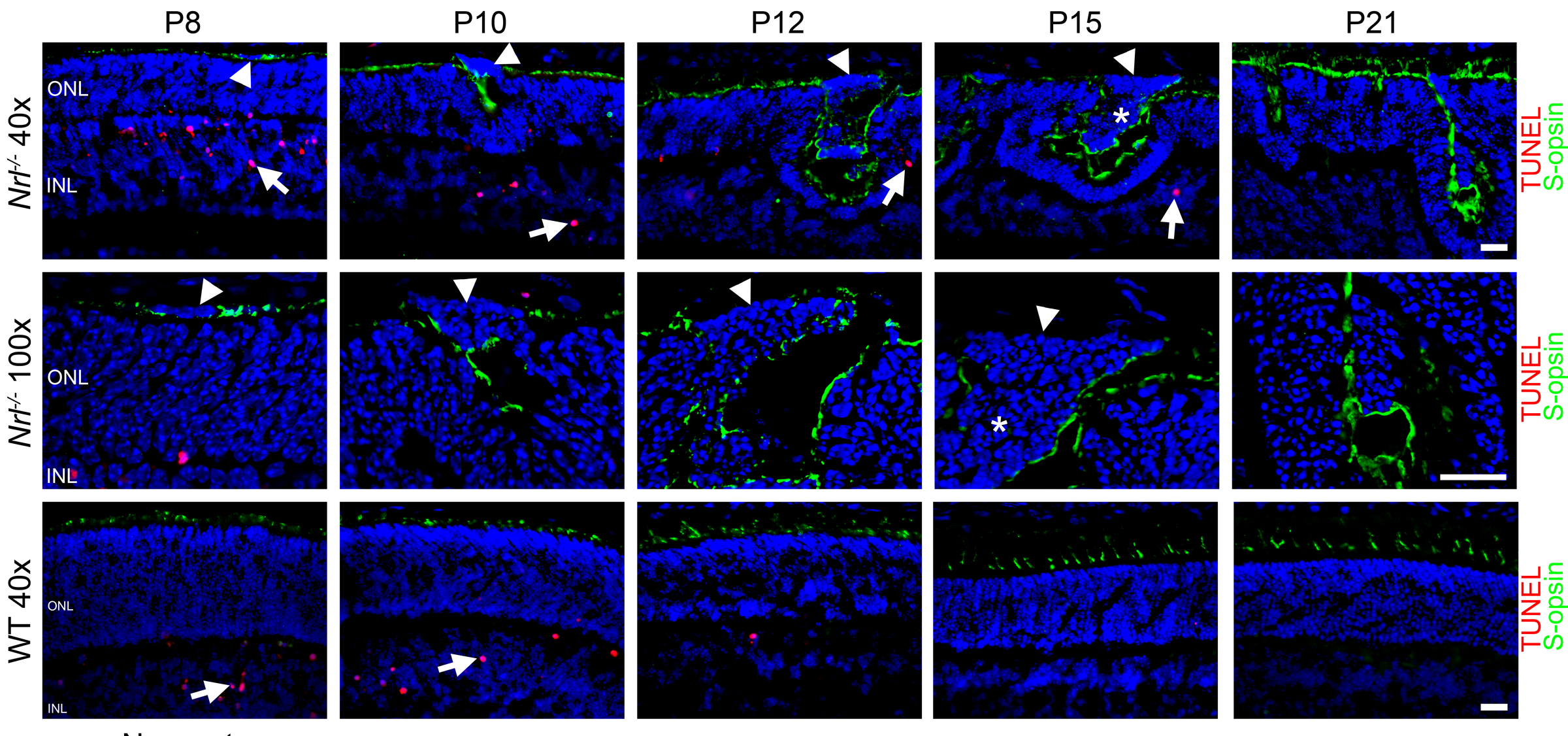

Supplement: Figure S3 — Aberrant photoreceptors are not cleared through apoptosis. A. TUNEL staining (red) was performed and followed by staining of OSs using S-opsin antibody (green). TUNEL positive cells can be seen in the INL as part of normal retinal development (arrows). All aberrant photoreceptors are negative for TUNEL staining at all time points examined (arrowheads). As previously noted, at P15 both nascent rosettes (containing aberrant photoreceptors) and mature rosettes are observed. We hypothesized that aberrant photoreceptors at this timepoint would be the most likely to be undergoing apoptosis if cell death is the mechanism underlying transition from the nascent rosette phenotype to the mature rosette phenotype. However, we did not detect any apoptotic cells in the nascent rosettes remaining at P15 (asterisks). ONL: outer nuclear layer, INL: inner nuclear layer. Scale bar, 20 µm. (TIF) [file pone.0032484.s003.tif]
